# The effect of IL-1 blockers on exertional leg pain in familial Mediterranean fever patients: an exploratory study

**DOI:** 10.1093/rheumatology/keag160

**Published:** 2026-04-22

**Authors:** Amit Druyan, Avi Livneh, Eitan Giat, Ilan Ben-Zvi, Merav Lidar

**Affiliations:** Rheumatology Unit, Sheba Medical Center, Ramat Gan, Israel; Gray Faculty of Medical and Health Sciences, Tel Aviv University, Tel Aviv, Israel; Department of Medicine F, Sheba Medical Center, Ramat Gan, Israel; Rheumatology Unit, Sheba Medical Center, Ramat Gan, Israel; Gray Faculty of Medical and Health Sciences, Tel Aviv University, Tel Aviv, Israel; Rheumatology Unit, Sheba Medical Center, Ramat Gan, Israel; Gray Faculty of Medical and Health Sciences, Tel Aviv University, Tel Aviv, Israel; Rheumatology Unit, Sheba Medical Center, Ramat Gan, Israel; Gray Faculty of Medical and Health Sciences, Tel Aviv University, Tel Aviv, Israel; Department of Medicine F, Sheba Medical Center, Ramat Gan, Israel; Rheumatology Unit, Sheba Medical Center, Ramat Gan, Israel; Gray Faculty of Medical and Health Sciences, Tel Aviv University, Tel Aviv, Israel

**Keywords:** familial Mediterranean fever, IL-1, leg pain, IL-1 blockers, IL-1 receptor antagonist, quality of life

## Abstract

**Objective:**

Exertional leg pain (ELP) is a distinctive musculoskeletal manifestation of familial Mediterranean fever (FMF), associated with severe disease and chronic inflammation. Despite adequate control of disease activity with colchicine, ELP often persists and contributes to disability, creating a serious unmet therapeutic need. This exploratory study evaluated the efficacy of IL-1 blockers in alleviating ELP in colchicine-resistant FMF (CR-FMF) patients.

**Methods:**

Twenty-seven CR-FMF patients treated with IL-1 blockers (23 canakinumab, four anakinra) at the Sheba Medical Center FMF clinic were included. Patients retrospectively assessed ELP severity and quality of life (QOL), using a 0–10 visual analogue scale (VAS), before and during IL-1 blocker treatment. The primary end point was reduction in ELP severity, measured by change in VAS pain score.

**Results:**

The cohort exhibited a severe phenotype (mean pre-treatment annual attack rate 50.69 ± 35.4; 55.5% M694V homozygous; 11% with amyloidosis). Following IL-1 blockade, 52% (14/27) of patients reported improvement in ELP. Mean pain scores decreased by 3.0 ± 3.68 points (*P* = 0.0003), and QOL scores improved by 3.07 ± 3.73 points (*P* = 0.0002). Residual ELP severity correlated strongly and negatively with QOL under biological treatment (*r* = −0.73, *P* < 0.0001)

**Conclusion:**

IL-1 blockers provide meaningful benefit in approximately half of patients with FMF-related ELP, supporting their role as a therapeutic option for this refractory manifestation. However, the persistence of ELP in a substantial proportion of patients underscores the need for additional therapeutic strategies to address this disabling manifestation.

## Graphical abstract

**Graphical Abstract:**
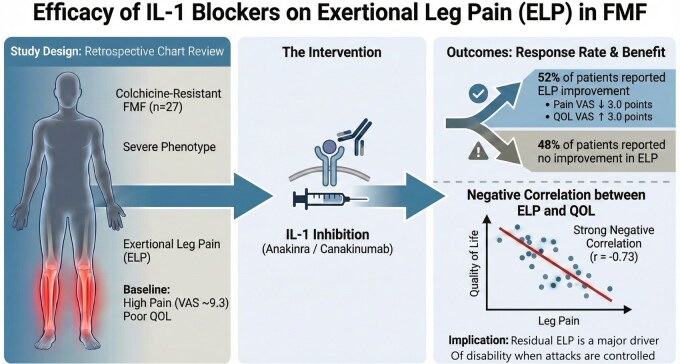

Rheumatology key messages

- IL-1 blockade reduces exertional leg pain (ELP) in approximately half of patients with colchicine-resistant familial Mediterranean fever.
- Under IL-1 inhibition QOL shows a negative correlation with residual ELP.
- Persistent ELP in many patients highlights the need for additional therapeutic options.

## Introduction

Familial Mediterranean fever (FMF), the most prevalent monogenic autoinflammatory disease, is characterized by recurrent episodes of fever and serositis. While classic FMF attacks, such as peritonitis, pleuritis and synovitis are well-described, and typically responsive to colchicine, exertional leg pain (ELP) remains less clearly defined.

Defined in the Tel-Hashomer criteria [1] but overlooked in earlier reports [2, 3], ELP was subsequently characterized by a controlled provocation trial [4]. ELP was shown to be triggered by prolonged standing or sitting, affecting the calves and feet, and often extending to the ankles, knees and hips. Symptoms persisted for hours after rest, and were frequently accompanied by swelling, redness and tenderness. Importantly, colchicine prophylaxis appeared ineffective or less effective as compared with attack control [4, 5]. Accordingly, EULAR recommends only symptomatic relief with NSAIDS for ELP management [6].

Consistent with colchicine refractoriness, ELP is not a minor complaint, but is associated with a severe FMF phenotype, ongoing inflammation, homozygous M694V genotype and an increased risk of amyloidosis and organ damage [7, 8]. These findings suggest that the presence of ELP identifies patients requiring more intensive treatment approach.

For individuals with colchicine-resistant FMF (CR-FMF), IL-1 blockers, such as anakinra and canakinumab are the standard of care. While their efficacy in reducing febrile attacks is well established [9, 10], their specific impact on ELP has not been systematically investigated. This exploratory study therefore aims to evaluate the efficacy of IL-1 blockers in alleviating ELP in patients with severe FMF, addressing a key gap in the management of this refractory manifestation.

## Patients and methods

### Setting

The study was conducted at the National FMF Clinic at Sheba Medical Center. Patient information is documented in a structured computerized registry. Diagnosis is clinical, based on Livneh’s criteria [1]. Standard treatment consists of colchicine (1–3 mg/day). In patients with resistance, intolerance or AA amyloidosis, IL-1 blockers are prescribed alongside maximally tolerated colchicine.

### Study population

We enrolled 27 CR-FMF patients. All were treated with IL-1 blockers (23 canakinumab, four anakinra), in addition to colchicine at a maximally tolerated dose. Prior to initiation of IL-1 blockers, all patients had documented ELP and uncontrolled inflammatory attacks. ELP was defined as bilateral pain in the calf or/and ankle or/and foot, with or without oedema, occurring after relatively minor effort and remitting with rest.

#### Inclusion criteria—eligible patients met the following criteria

1. Diagnosis of FMF according to Livneh’s criteria [1].
2. Age ≥18 years.
3. Documented resistance or intolerance to colchicine.
4. Treatment with IL-1 blockers for >3 months at the time of inclusion.
5. History of ELP prior to initiation of biological treatment.

#### Data extraction and definitions

Baseline characteristics were retrieved from the computerized registry. Variables included: sex, age at diagnosis/onset, age at inclusion, age at IL-1 blocker initiation, duration of Il-1 blocker treatment at inclusion, type of IL-1 blocker used (anakinra or canakinumab), M694V homozygosity status, biopsy-proven amyloidosis, attack frequency while on colchicine (number of attacks during the year preceding IL-1 blocker initiation), frequency of attacks under IL-1 inhibition (number of attacks during the year preceding inclusion, or extrapolated if IL-1 blocker treatment duration was less than one year), colchicine dose, attack types during the disease course (abdominal, articular, chest, fever-only, erysipelas like erythema), attack-free CRP levels (last documented attack-free CRP levels before and during IL-1 blockade) and disease severity by Mor severity score [11].

The study was approved by the Sheba Medical Center Helsinki Committee (8703–21-SMC), which waived the requirement for written informed consent as the committee determined that participants’ voluntary completion of the questionnaires constituted implied consent.

### Historical controls

To provide a benchmark for disease severity in the study group, baseline characteristics were compared with a previously published cohort of 99 FMF patients with ELP, who had not received biologic treatment [8]. This historical cohort originated from the same centre and database, ensuring consistency and reliability in demographic and clinical comparisons.

### Assessments

The primary end point was reduction in ELP severity, measured by change in visual analogue scale (VAS) pain score.

Patients retrospectively evaluated two parameters using a 0–10 VAS, comparing their condition before (while on colchicine monotherapy) and during IL-1 blocker therapy:

- ELP severity: 0 = no pain; 10 = worst pain.
- Quality of life (QOL): 0 = severe disability; 10 = no disability.

### Data evaluation and statistical analysis

Changes in ELP severity, QOL, CRP levels and annual attack rate before and during IL-1 blocker treatment were analysed using paired *t*-tests. The proportion of patients showing improvement in ELP and achieving normal CRP levels following IL-1 blockade was compared with a null hypothesis of no response using a *χ*^2^ test. Continuous variables were compared between cohorts using independent *t*-tests based on published means and standard deviations, and categorical variables were assessed using *χ*^2^ tests based on reported proportions and patient counts. A complete case analysis was performed; no data imputation was applied. The association between ELP severity and QOL under IL-1 blocker treatment was evaluated using linear regression. A two tailed *P*-value <0.05 was considered statistically significant.

## Results

### Patient characteristics

Table 1 presents the demographic and clinical characteristics of the 27 patients in the study cohort and 99 patients in the historical cohort. The study group exhibited a markedly severe phenotype, with 85% classified as severe disease according to Mor criteria [11]. This was reflected by a high pre-treatment attack rate (∼50/year), a high prevalence of amyloidosis (11%) and intensive colchicine use, with a mean daily dose of 2.6 mg. Consistent with a severe phenotype, the majority were of North African Jewish descent (64%), followed by other Jewish (Iraqi, Iranian, Syrian, Ashkenazi), Arab (*n* = 1) and Druze (*n* = 1) origins. Although the two cohorts were broadly comparable across most parameters (Table 1), the study group demonstrated a more severe disease, with several-fold higher attack frequency, higher colchicine dose and higher disease severity score.

**Table 1:** Characteristics of study cohort and historical controls.

Characteristic	Study cohort (*n* = 27)	Historical ELP cohort^a^ (*n* = 99)	*P*
Male gender, *n* (%)	12 (44.4)	58 (58.6)	0.18
Age at inclusion (mean ± S.D., years)	43.99 ± 13.2	32.7 ± 12.4	**<0.0001**
Age at onset (mean ± S.D., years)	9.51 ± 7.69	9.5 ± 9.0	0.99
Age at diagnosis (mean ± S.D., years)	16.46 ± 12.22	14.2 ± 10.9	0.3540
Age at biological treatment (mean ± S.D., years)	42.07 ± 12.9	N/A	
Duration of IL-1 blocker treatment at study inclusion (mean ± S.D., months)	23.07 ± 15.24	N/A	
M694V homozygosity, *n* (%)	15 (55.5)	45 (45.5)	0.35
Amyloidosis, *n* (%)	3 (11)	3 (3)	0.08
Attacks/year on colchicine (mean ± S.D., *n*)	50.69 ± 35.4	8.1 ± 9.9	**<0.0001**
Attacks/year on IL-1 blockers (mean *n*)	9.16 ± 19.3	N/A	
Attack-free CRP on colchicine (mean ± S.D., mg/dcl)	1.81 ± 1.38	Not studied	
Attack-free CRP on IL-1 blockers (mean ± S.D., mg/dcl)	0.95 ± 1.59	N/A	
Colchicine dose (mean ± S.D., mg/day)	2.62 ± 0.51	1.4 ± 0.6	**<0.0001**
Abdominal attacks, *n* (%)	27 (100)	87 (87.9)	0.057
Leg arthritis attacks, *n* (%)	23 (85.1)	74 (74.7)	0.25
Pleuritic attacks, *n* (%)	18 (66.67)	48 (48.5)	0.094
Fever-only attacks, *n* (%)	15 (55.5)	35 (35.4)	0.057
Erysipelas-like erythema in shin, *n* (%)	6 (22.2)	14 (14.1)	0.31
Disease severity (0–6), mean ± S.D.	3.81 ± 1.21	2.0 ± 0.3	**<0.0001**

a Eshed *et al.* [12].

ELP: exertional leg pain; N/A: not applicable. Statistically significant *P* values are written in bold.

ELP in all patients was, by definition (section ‘Patients and methods’) and in accordance with real-world presentation, consistently bilateral, provoked by exertion, mostly prolonged standing or walking. Symptoms resolved after several h of rest, typically overnight. Owing to this characteristic clinical pattern, additional imaging or laboratory investigations specifically targeting ELP were seldom performed, outside routine assessments. These evaluations were generally unremarkable, except for inflammatory markers, such as CRP (Table 1). Notably, despite the overall severe disease phenotype, none of patients had a history of protracted febrile myalgia.

### Effect of treatment on ELP

IL-1 blockade substantially reduced the mean annual attack rate from ∼50 to 9 (mean reduction 42.45 ± 32.69; *P* < 0.0001). The decline in attack frequency was accompanied by normalization of elevated attack-free CRP levels in 50% of the patients (*P* < 0.0001, compared with a null hypothesis of no improvement). Notably, the absolute reduction in CRP did not reach statistical significance (*P* = 0.08), likely due to wide variability (Table 1).

**Figure 1:**
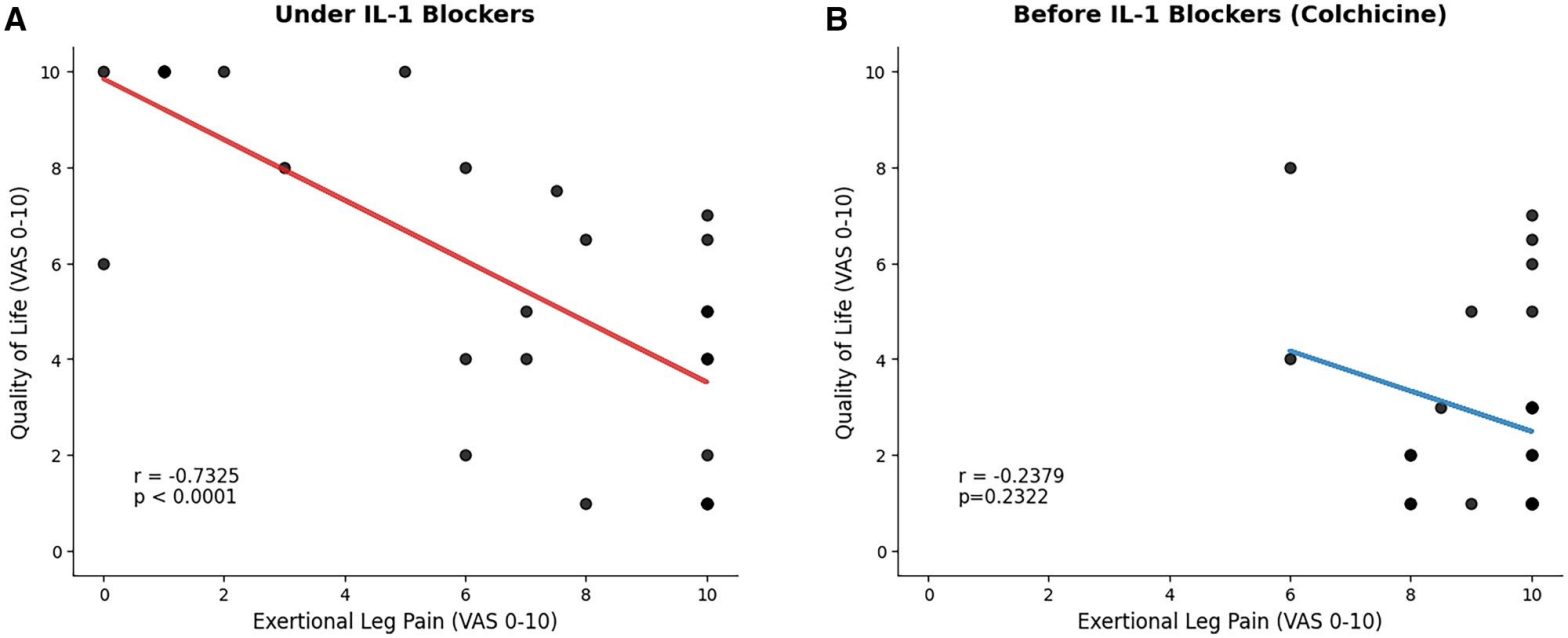
Impact of IL-1 blocker treatment on the relationship between ELP on QOL. Linear regression analysis of patient-reported VAS scores (0–10). (**A**) Under IL-1 blocker treatment, a significant negative correlation observed between ELP severity and QOL (*r* = −0.7325, *P* < 0.0001). (**B**) Prior to biological treatment (colchicine only), no significant correlation was detected (*r* = −0.2378, *P* = 0.23). ELP—exertional leg pain, QOL—quality of life, VAS—visual analogue scale

The primary end point—reduction in ELP severity—was achieved in 52% of patients (*P* < 0.005, vs no improvement hypothesis). Mean VAS pain scores decreased by 3.0 ± 3.68 points (9.27 ± 1.21–6.27 ± 3.76; *P* = 0.0003). Concurrently, QOL scores improved by 3.07 ± 3.73 points (2.79 ± 2.13–5.87 ± 3.24; *P* = 0.0002). Linear regression analysis demonstrated a strong inverse correlation between the ELP severity and QOL during IL-1 blocker treatment (*r* = −0.7325, *P* < 0.0001, Fig. 1A). Notably, prior to IL-1 blockade, when colchicine was the sole treatment, no such relationship was observed (Fig. 1B). This pattern suggests that the impact of ELP on QOL had previously been masked by high disease activity in other domains and became apparent only after IL-1 inhibition effectively controlled the additional manifestations.

## Discussion

This exploratory study demonstrates that IL-1 blockers provide significant relief for approximately half of FMF patients with ELP. However, despite effective suppression of febrile attacks, ELP remains a refractory manifestation for a substantial proportion of individuals.

While 52% of patients reported improvement, this result should be interpreted with caution. Nearly half of the cohort continued to experience ELP despite effective suppression of febrile attacks, and even among responders, improvement was frequently partial, rather than complete. Thus, despite advancement made in ELP management with IL-1 blockers, ELP continues to pose a significant therapeutic challenge.

Several factors may contribute to the lack of improvement in ELP among some patients, one of which is the marked FMF disease severity in our study subset. Although ELP marks a severe phenotype [8], our biologic-treated cohort exhibited an even greater disease burden than historical controls, consistent with patients requiring biologic therapy. This was reflected in markedly higher attack rates, a tendency towards increased prevalence of amyloidosis, greater colchicine requirements and predominance of the homozygous M694V genotype. The limited response to IL-1 blockade may therefore relate to this extreme severity, rather than insufficient drug efficacy. Milder ELP cases may respond more favourably to IL-1 inhibition.

The variable response to IL-1 inhibition suggests a multifactorial pathophysiology. Previous MRI studies identified occult spondyloarthritis and enthesopathy in ELP patients, features typically mediated by TNF and IL-17 rather than IL-1 [12]. Additionally, MRS findings of altered muscle metabolism suggest an underlying metabolic myopathy distinct from classic inflammation [13]. These IL-1-independent mechanisms may explain the limited efficacy of IL-1 blockade in non-responders.

A notable finding of this study is the emergence of a strong negative correlation between ELP severity and QOL under biological treatment (*r* = −0.7325), a relationship that was absent prior to this therapy. Before IL-1 blockade, QOL was likely dominated by frequent febrile attacks (∼50/year), masking ELP’s specific contribution. Once attacks were suppressed, the impact of ELP on daily functioning became apparent. This observation highlights that effective FMF management requires attention beyond attack control, as persistent ELP remains a major determinant of disability and prevents full restoration of QOL. This aligns with previous findings, identifying ELP as a predictor of unsuccessful IL-1 blocker withdrawal in patients who otherwise achieved attack control [14].

This study has several limitations that must be acknowledged. First, its retrospective design introduces inherent biases, particularly recall bias, in grading pain severity and QOL prior to IL-1 blocker treatment. However, in the context of ELP, this limitation may be less pronounced, as patients actively try to avoid triggering these painful episodes and therefore tend to remember these events and their functional impact more vividly.

Second, because the study required a unique subset of FMF patients (colchicine-resistant individuals with ELP), the sample size (*n* = 27) is relatively small. This limited our ability to identify clinical or genetic predictors of response to IL-1 blockers, and we could not statistically determine why some patients responded favourably while others remained refractory.

Third, several features essential for a more complete characterization of ELP were unavailable. This was partly due to the retrospective design of the study (e.g. missing data on age at ELP onset) and partly because routine clinical practice does not typically include evaluations aimed at better elucidating ELP, such as muscle enzyme testing or imaging studies. A prospective study designed to define the clinical profile of ELP in FMF would be valuable to address these gaps.

Nonetheless, the small sample size is consistent with the exploratory nature of the study, and the unmet need for effective treatment of ELP is addressed here for the first time by systematically evaluating the effect of IL-1 blockade. Given the absence of prior data, our findings provide a foundation for future prospective studies with larger cohorts which will be essential for validating our results and identifying predictors of treatment response.

## Conclusion

IL-1 blockers offer clinical benefit to approximately half of FMF-ELP patients. However, the lack of response observed in the remaining patients underscores the complex pathogenesis of ELP, which likely involves several inflammatory pathways as well as non-inflammatory metabolic mechanisms. The particularly severe FMF phenotype of the study cohort may have further contributed to treatment resistance. Consequently, ELP remains with a significant unmet need in FMF care, highlighting the necessity for further research, into both IL-1 dependent and IL-1 independent pathways to develop more effective therapies for all patients.

## Data Availability

The data underlying this article will be shared on reasonable request to the corresponding author.
